# The Scientific Basis and Advantage of Human Experiential Assessment in the quality control of Chinese Herbal Medicines exampling as Schisandrae Chinensis Fructus

**DOI:** 10.1038/s41598-018-23619-5

**Published:** 2018-04-09

**Authors:** Yongfeng Zhou, Dingkun Zhang, Haotian Li, Haizhu Zhang, Jixiang Fang, Yanqin Ma, Ping Zhang, Jiabo Wang, Xiaohe Xiao

**Affiliations:** 1 China Military Institute of Chinese Medicine, 302 Military Hospital, Beijing, 100039 China; 2 College of Pharmacy, Chengdu University of Traditional Chinese Medicine, Chengdu, 611137 China

## Abstract

Experiential quality assessment(EQA) is an important sensory analysis for judging herbal quality grades. Because of the high empirical utility of expert experience, the consistency, science and inheritance of such experience are continuously in dispute. To explore the scientific evidence for this subjective method, we designed a Delphi expert investigation coupled with chemical analysis to evaluate the quality of Schisandrae Chinensis Fructus (SCF). Initially, 13 experts were invited to independently evaluate the grades of 11 batches of SCF. After screening the consistency and repeatability of the evaluation results, typical samples of all quality levels were identified. Seven significant physical characters were detected; colour and size were found to be the key parameters for identifying SCF quality. Based on this correlation, a decision tree model was ultimately established and converted to a quality evaluation card. Over 80% consistency in a novice test demonstrated the technical advantages and application characteristics of the model. Further correlation analysis revealed that EQA quality grades of SCF were positively correlated to the content of polysaccharides and polyphenols, while negatively correlated to the content of lignans. Biological activities were also approving it. In summary, our study proves that subjective EQA is consistency, repeatability and could be inherited.

## Introduction

With the advancement of detection technology, the quality evaluation of Chinese herbal medicine (CHM) has entered the era of chemical analysis. Characteristic fingerprinting and multi-component content determination provide good qualitative and quantitative evaluation models. However, regardless of whether HPLC^1^ or LC-MS^2^ technology is used, doing so requires specific instrumentation and sample preparation procedures, and it is difficult to meet the needs of on-site, rapid and nondestructive identification.

In the field of CHM, pharmacognosy experts and experienced masters customarily judge the quality of herbs based on features such as color^3^, smell^4,5^, flavour^6^, size^7^, and weight^8^. This method is named experiential quality assessment (EQA). EQA usually classifies CHM into several grades, which are indicative of different levels of quality. This method has prominent advantages, such as being relatively simple, easy to implement, and nondestructive. However, the method is accompanied by obvious shortcomings. On one hand, EQA is highly dependent on the experience of experts, and beginners do not easily master the approach. Moreover, with the gradual death of masters, EQA is facing an increasingly serious inheritance crisis. On the other hand, EQA provides an empirical summary of previous practice, which is established based on the correlation between the appearance and treatment effect of CHM. However, the passing of time requires us to explore the scientific results of this subjective method.

Schisandrae Chinensis Fructus (SCF) stems from the dry ripe fruits of *Schisandra chinensis* (Turcz.) Baill of Magnoliaceae plant. SCF is considered a wonder drug because it features five different tastes. The pulp is sour and sweet, the kernel is bitter and pungent, and both parts are salty. Based on this abundance of flavours, our ancestors gradually recognized SCF’s antitussive, antidiarrheal, antidipsetic, and tranquilization effects. In the 1950s, a young Chinese doctor used a SCF decoction to treat a liver disease patient with severe insomnia. After a period of treatment, the ALT and AST levels of the patient decreased significantly, and the hepatoprotective effect of SCF was discovered by accident. Later, natural medicine scholars isolated several dibenzocyclooctene lignans from SCF, including schizandrol and schizandrin, and developed new hepatic protectors. The hepatoprotective^9–11^, anti-inflammatory^12,13^, anti-cancer^14^, and anti-oxidation^15^ effects have since attracted wide attention and application.

Today, SCF is an internationally famous traditional natural medicine. As a fruit herb, SCF has many distinctive characteristics, such as its size, colour, and taste. Different experts determine the quality grade of SCF according to their own experience, and there is no uniform standard. The literature also reveals variations in how to judge the quality grade of SCF. For example, in the book *Shen Nong’s Herbal*, the quality of SCF was graded by size and colour, while in *76 Specifications of Medicinal Materials*, the herb was mainly judged by colour and taste. Therefore, how can the appropriate indicators for evaluating the quality of SCF be chosen scientifically? Is there an intrinsic relationship between different evaluation indicators and their chemical components? According to Ch.P2015, schisandrol A is used for the quality control of SCF in the Chinese Pharmacopoeia, but the selected indexes are too simple and show low specificity such that they cannot truly reflect inherent quality. Therefore, how can we evaluate the quality of SCF more comprehensively?

In this study, different commodity grades of SCF were collected as study cases. The accuracy and reproducibility of TCM expert evaluations were investigated by a two-step survey. The main indicator of EQA and quantitative factors determined by experts were submitted to statistical analysis. The SPSS software was used to establish a decision tree model, and a quality evaluation card (QEC) was created based on the results. The cards were given to novices. The inheritance of EQA was proved by comparing the results obtained from TCM experts and novices. In addition, chemical constituents such as lignans, polysaccharides and polyphenols in different grades of SCF were analysed to reveal the scientific merit of EQA. Our research provides a novel study model of the inheritance and innovation of traditional empirical identification, which is of great interest to pharmacists and doctors. In addition, this study provides a quick and accurate method for determining how to inherit EQA, an intangible cultural heritage in TCM that is gradually disappearing.

## Results

### Delphi survey for experts and samples

**Figure 1:**
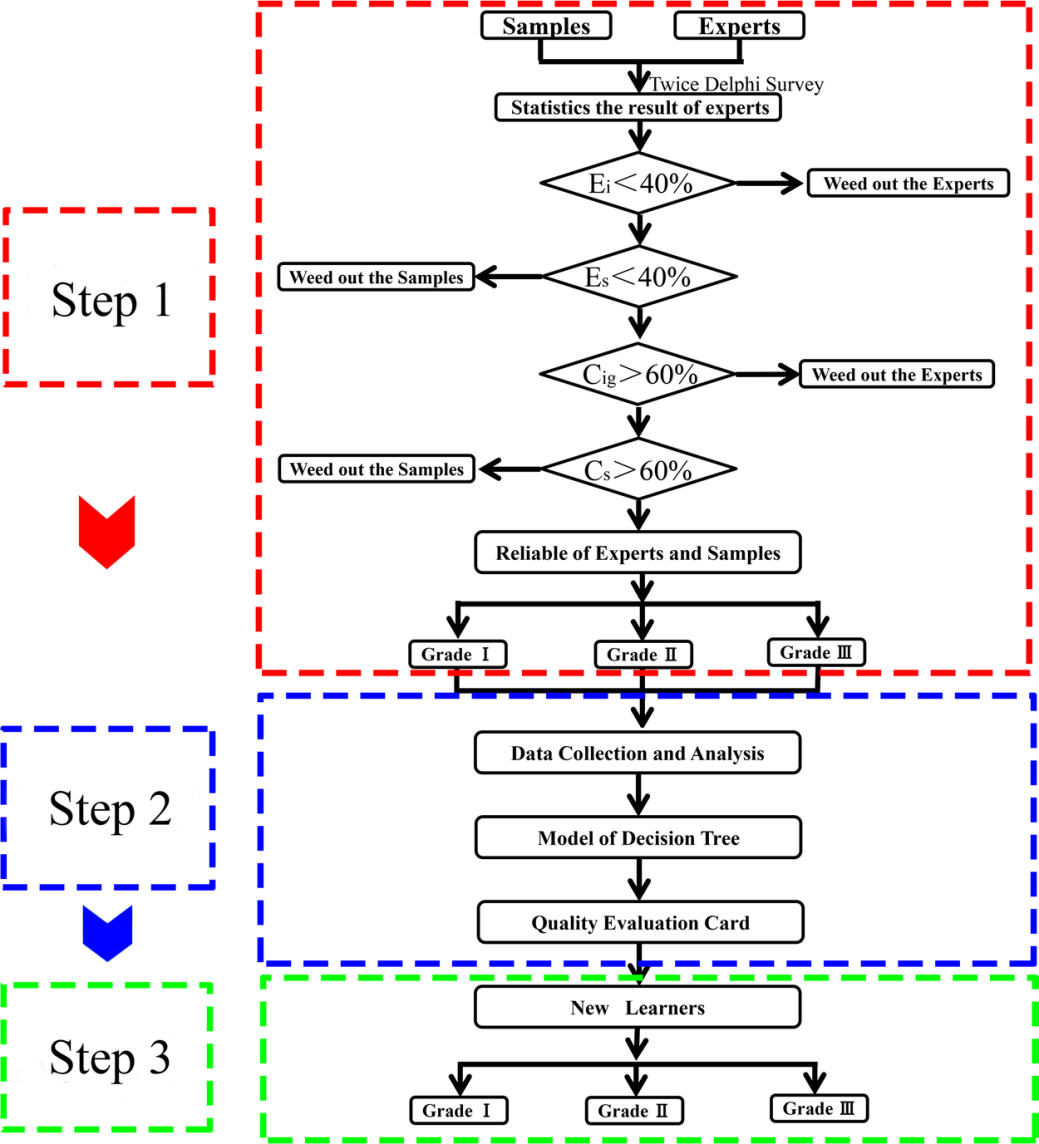
Flow chart of the method: step 1: Preliminary EQA for quality grade and unqualified experts and samples were weeded out; step 2: Model establishment and EQA card design; step 3: Validation of the EQA inheritability in novices. (This image was created by Dr. Zhou).

**Figure 2:**
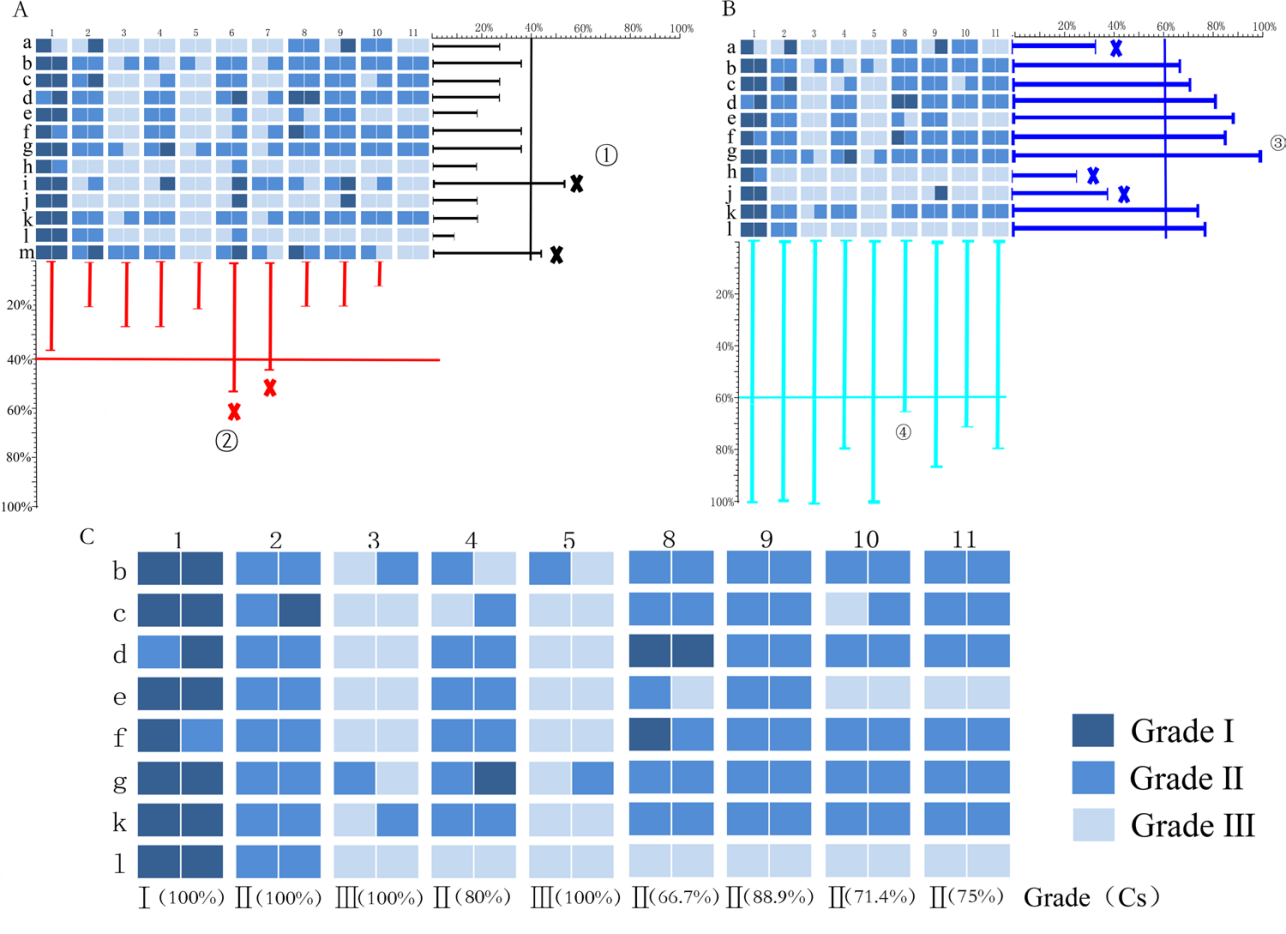
Preliminary screened results of samples and experts (**A**). Further screened results of samples and experts (**B**). Final screened results (**C**). X axis represents the samples and Y axis represents the experts. Different color represents the different grade of SCF; the darker the color, the higher the grade. (This image was created by Dr. Zhang).

A Delphi survey was used to judge the different grades of SCF and statistically analyse experts’ judgement. Evaluation parameters E_i_, E_s_, C_ig_ and C_s_ were calculated by equations 1, 2, 3 and 4. The selection criteria and flow chart are shown in Fig. 1. Experts and samples with E_i_ and E_s_ values above 40% were rejected during the preliminary screening (Fig. 2A). Then, other experts and samples with C_ig_ and C_s_ values less than 60% were rejected during the second screening (Fig. 2B). Based on the screening indicators, we excluded five experts, namely, a, h, i, j, m, and two samples, No. 6 and No. 7. The screening results are shown in Fig. 2C.

### Analysis of factors of experts and quality grade

**Figure 3:**
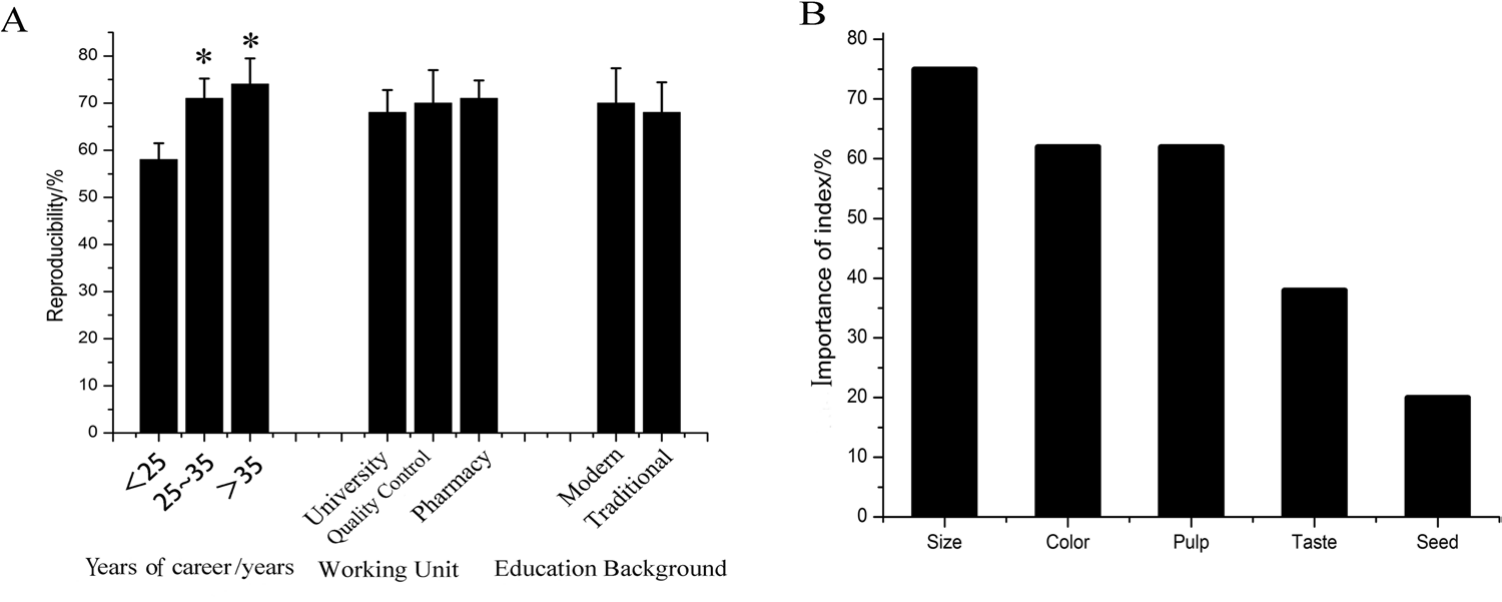
Relationship between the reproducibility of experts and career years, working unit and education background (**A**). The result shows that the reproducibility has significant correlation with career years. The longer the career years, the higher the reproducibility. But the working unit and education background have no significant correlation with the reproducibility. Index importance of sensory evaluation (**B**), which was calculated by Equation 3. (This image was created by Dr. Zhou).

The correlation between the Delphi survey results and experts’ number of career years, nature of work and educational background was analysed. These results showed that the number of career years was significantly correlated with the accuracy of the experts’ judgements (Fig. 3A), with longer careers yielding higher accuracy. However, no evidence indicated that the experts’ nature of work and educational background affected the accuracy of the evaluation results.

Based on the importance of the index calculated by equation 5, we analysed the result pertaining to the importance of the evaluation index determined by experts (Fig. 3B). These results showed that the most importance indexes of sensory evaluation were size, pulp and colour. With respect to plant tissue structure, SCF is composed of pulp, kernel and testa, and plant size is positively related to the pulp fraction. Therefore, the three indexes selected by experts can be summarized by size and colour.

### Results of decision tree and quality evaluation card (QEC)

**Figure 4:**
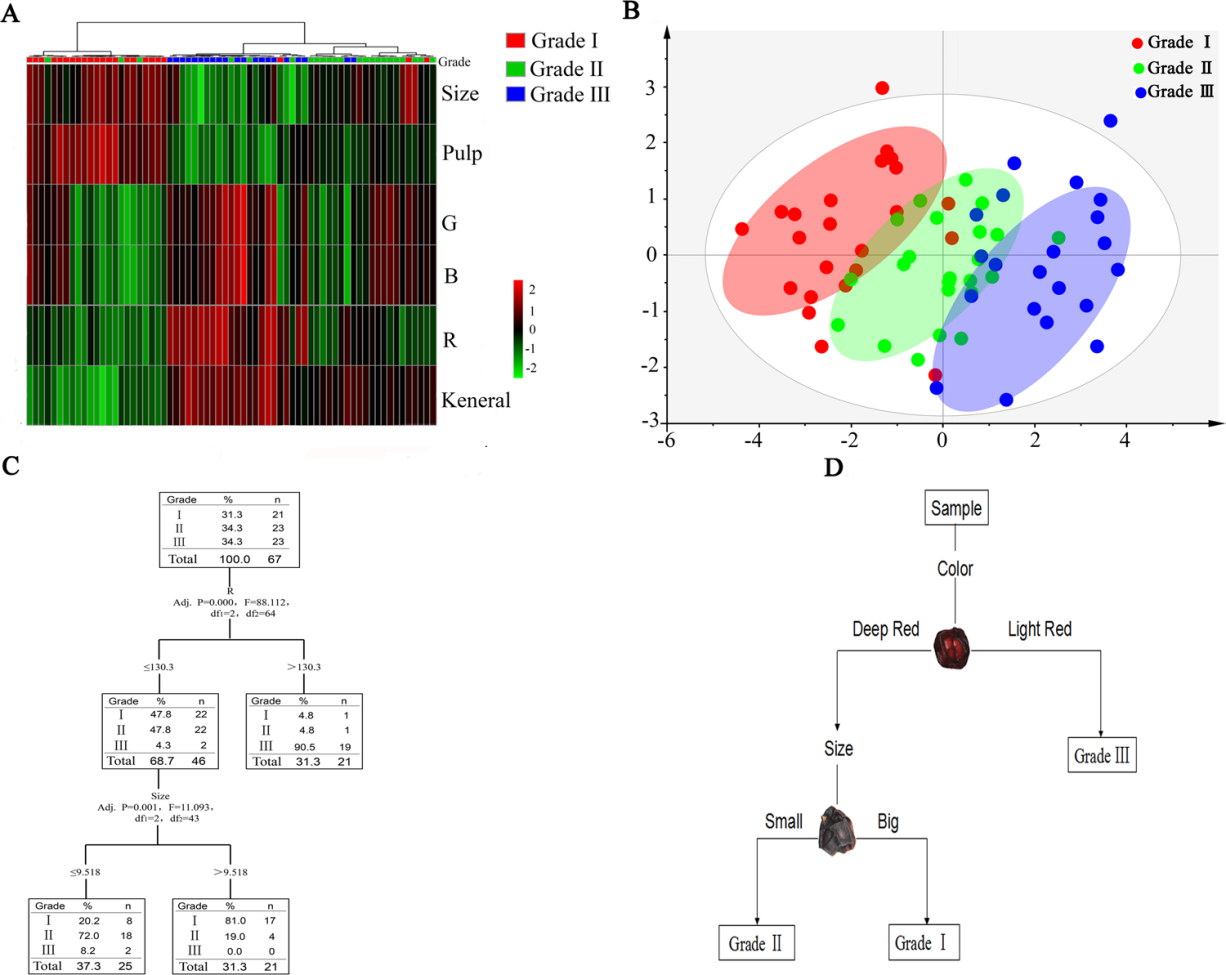
CA Results of SCF samples (**A**), which was calculated on the physical parameters in Table 1. PCA Results of SCF samples (**B**), and different grades of SCF can be obviously discriminated. Decision tree model (**C**), which was built on the character parameters. Using this model, we can judge the grade of SCF with two steps. The first step was to determine the color of SCF; if the color R was more than 130.3 and this sample was most likely to be grade III; if it was less than 130.3, we need to judge the grade by size. If the size was greater than 9.518 mm, it may be grade I, or it may be grade II. Quality evaluation card (**D**), which was designed based on the decision tree above and color and size were the same as the actual. (This image was created by Dr. Zhang).

According to Fig. 3B, different indexes were collected from the different grades of SCF. Cluster analysis (CA) and principal component analysis (PCA) results are shown in Fig. 4A,B. These results illustrate that different grades of SCF could be significantly distinguished, and size and colour were the main variables allowing for grouping. To identify the grade of SCF quickly, a decision tree (Fig. 4C) was created using the SPSS software. The decision tree was used to evaluate the grade of SCF in two steps: the first step was to determine the colour of SCF; if a sample’s R colour value was greater than 130.3, the sample was most likely grade III; values less than 130.3 required the grade to be judged based on size. If the sample size was greater than 9.518 mm, grade I was likely, or if not, grade II was likely. A quality evaluation card (Fig. 4D) was designed based on the decision tree model, and the colour and size were the same as the those of actual samples.

### Validation of QEC accuracy

**Figure 5:**
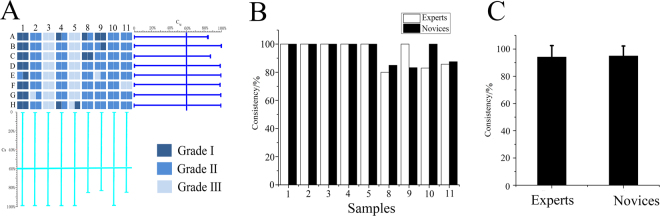
Results of novices judged the grade of SCF using QEC card (**A**). Consistency of evaluation results between novices and experts (**B**). (This image was created by Dr. Zhou).

Thirteen inexperienced students were given the card and taught how to use it. The students used the card to identify the grades of the 9 batches of SCF, and the results were compared with those of the experts (Fig. 5A,B). The results show that the consistency among the students was above 80% and better than that among the experts. These result indicate that the EQC can be used to quickly and efficiently identify the grades of SCF and is simple, convenient, and easy to study and inherit.

### Correlation analysis between chemical composition and traditional efficacy

**Figure 6:**
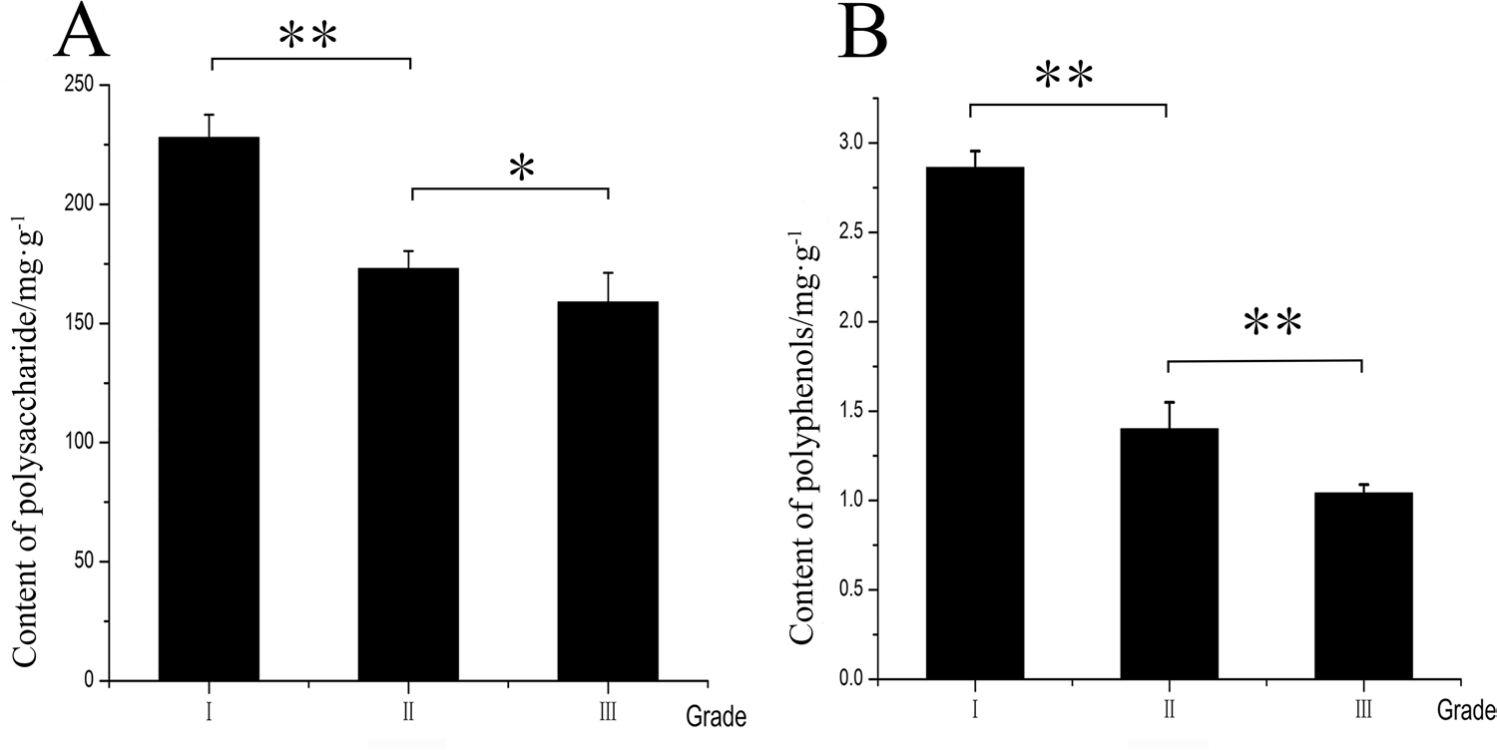
Contents of polysaccharide in different part of SCF (**A**). Contents of polyphenols in different part of SCF (**B**). (This image was created by Dr. Zhou).

SCF is mainly used as an anti-aging agent and antioxidant in TCM. Modern studies have shown that the main active ingredients in SCF are polysaccharides^16^ and polyphenols^17^. In this study, the contents of polysaccharides and polyphenols in the different grades of SCF were determined. The results showed that the contents of polysaccharides and polyphenols in the different grades of SCF varied (Fig. 6A,B). Generally, high contents of polysaccharides and polyphenols were correlated with high quality grades.

### Correlation analysis between chemical composition and modern efficacy

**Figure 7:**
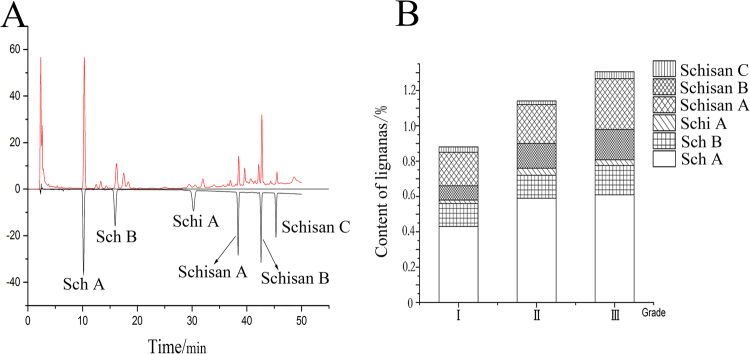
HPLC chromatogram of SCF, the above one was sample solution, and the below one was mixed references solution (**A**). Content of lignans in different part of SCF (**B**). (This image was created by Dr. Zhou).

Currently, SCF is mainly used as an anti-inflammatory and hepatoprotective, and the main chemical component is lignans^11,18^. In this study, the content of lignans in different grades of SCF was determined. The results showed that the content of lignans varied among different grades of SCF, and there was a negative correlation between the quality grade of SCF and the content of lignans (Fig. 7A,B). Generally, a low content of lignans led to a high quality grade.

**Figure 8:**
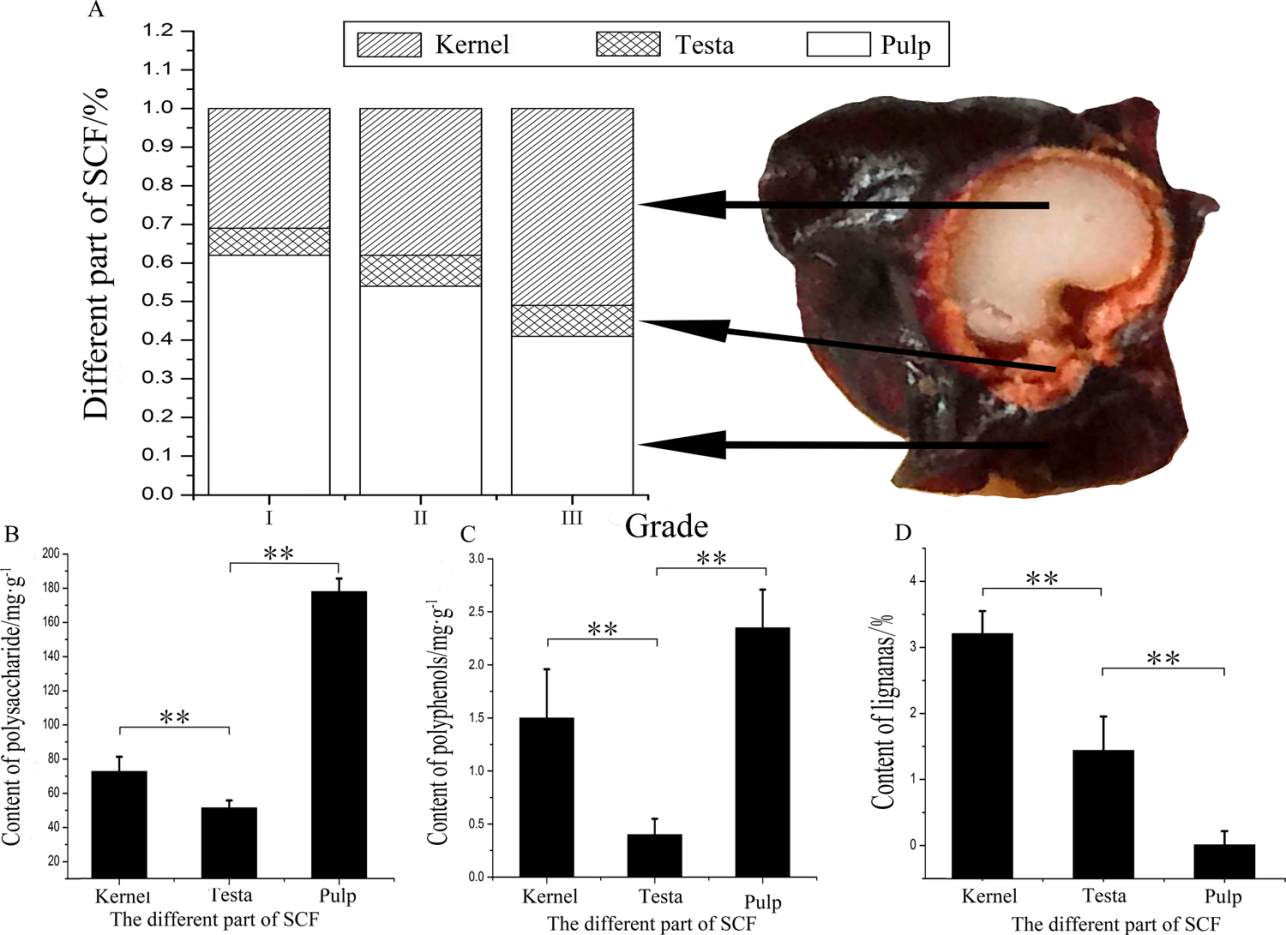
The structure and distribution of chemical constituents of SCF. The structure diagram and content of different part of SCF (**A**). The content of polysaccharide in different part of SCF (**B**). The content of polyphenols in different part of SCF (**C**). The content of lignans in different part of SCF (**D**). (This image was created by Dr. Zhang).

To explain this phenomenon, the structural composition of SCF was dissected. The results showed that SCF was composed of three parts: pulp, testa and kernel (Fig. 8A). However, the chemical contents of the three parts clearly varied. Polysaccharides and polyphenols were mainly distributed in the pulp, while lignans were mainly concentrated in the testa and kernel (Fig. 8B–D).

### Biological activity verification

**Figure 9:**
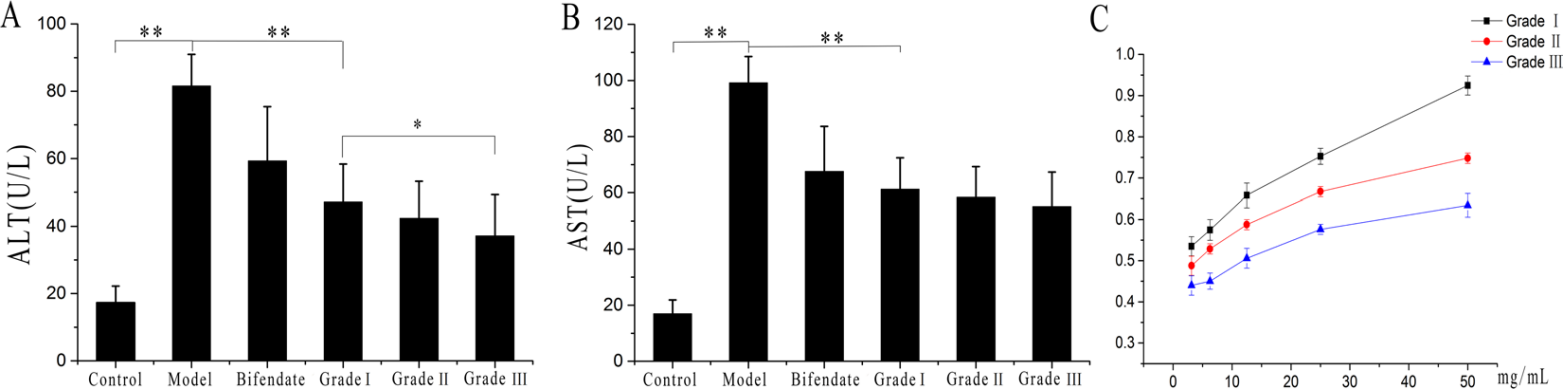
The biological activity of different grade of SCF. The effect of different grade of SCF to reduce the ALT in the serum (**A**). The effect of different grade of SCF to reduce the AST in the serum (**B**). The radical scavenging activity of different grade of SCF (**C**). (This image was created by Dr. Zhou).

To verify the differences in biological activity among the different grades of SCF, an *in vivo* liver injury model based on CCl_4_ was established to verify the hepatoprotective efficacy of the different grades of SCF, and the antioxidant capacity of the different grades of SCF was verified by DPPH radical scavenging *in vitro*. The results of the experiment show that the different grades of SCF can reduce ALT and AST level in mice with liver injury. Grades I and III showed significant differences in their ability to reduce ALT levels. No significant difference was observed with respect to AST levels but we also can find the reduce trend in different grade of SCF. The reason may be that the differences in the chemical contents of the different grades of SCF selected for this experiment are limited and are difficult to get the significant differences considered in pharmacodynamics. The abilities of the different grades of SCF to scavenge free radicals also varied. Grade I SCF showed the higher antioxidant capacity, while grade III showed the lower. This experiment demonstrated that the bioactivities of different grades of SCF are different and further proved that the EQA of SCF is scientifically sound (Fig. 9A–C).

## Discussion

The Delphi method is a consensus methodology for assessing the extent of agreement (consensus measurement) and for resolving disagreements (consensus development)^19^. As a classical research method, the Delphi method has been widely used in various medical fields, such as in nursing^20^, health economics^21^ and epidemiology^22^. In view of the limitations of the Delphi method in the identification of herbs, for example, there is lack of ideological communication among experts, possibly leading to subjective one-sidedness. In this experiment, we focused gathered the opinions of experts through the Delphi method to evaluate the quality of CHM. To effectively avoid one-sided expert judgements, we reached a consensus and conducted an intersection analysis of the experts’ experience. The results show that the experts reached a good consensus on SCF grade.

The decision tree is a very important data mining algorithm that offers many advantages, such as high precision, ease of quantification and ease of comprehension. This method has already been widely used in preventive medicine, public health and clinical auxiliary diagnosis and with good effect^23,24^. In this study, we statistically analysed parameters such as size and colour of different grades of SCF classified by experts. The decision tree was created based on these physical parameters using the SPSS software. To make the decision tree simple and easy to use, we analysed and quantified the node information of the decision tree and converted it to a card. The EQC was given to novices to judge the different grades of SCF. A comparison of the results of novices and experts showed that the consistency among novices was greater than that among experts; thus, EQA can be used to quickly and accurately inherit the experience of experts using the card. Furthermore, the chemical composition of different grades of SCF was determined, and a correlation analysis between quality grade and chemical composition was performed. The results showed that the contents of polysaccharides and polyphenols in SCF were significantly positively correlated with quality grade, indicating that the use of EQA to judge the quality of SCF is scientifically sound.

In this study, traditional sensory evaluation of CHM and the Delphi method were combined to explore the scientific merit and heritability of EQA. The combined method is innovative, but some deficiencies of this study are noted. For example, only a correlation analysis between chemical composition and quality grade was carried out, but no biological effect was examined for verification. Moreover, the number of samples used in the experiment was limited; a larger sample size is required for further verification. In conclusion, this study provides a new model for the empirical identification of Chinese herbal medicine and provides an important reference for the inheritance and innovation of EQA.

## Materials

11 batches of SCF were collected from Heilongjiang province, Jilin province and Liaoning province, China. All samples were identified by Professor Xiaohe Xiao and the voucher specimens were deposited in China Military institute of Chinese Medicine, 302 Military Hospital of China. The information about the different batches of SCF was listed in Table 1.

**Table 1:** Physical parameters about the different batches of SCF

No.	Origin	Physical parameters						
		R	G	B	Size	Pulp	Testa	Kernel
1	Jilin Province	77	52	52	10.25	0.65	0.08	0.27
2	Jilin Province	92	58	54	8.67	0.51	0.07	0.42
3	Jilin Province	177	79	76	7.56	0.43	0.07	0.5
4	Liaoning Province	99	63	61	8.67	0.51	0.06	0.43
5	Liaoning Province	166	70	65	7.92	0.45	0.07	0.48
6	Liaoning Province	178	75	71	7.61	0.42	0.06	0.52
7	Liaoning Province	164	65	59	7.08	0.45	0.05	0.5
8	Heilongjiang Province	99.8	62	58.3	8.754	0.512	0.061	0.427
9	Heilongjiang Province	90.3	61.9	60.1	8.804	0.506	0.065	0.429
10	Heilongjiang Province	98.7	62.9	60.6	8.674	0.511	0.062	0.427
11	Heilongjiang Province	90.3	61.9	60.1	8.804	0.506	0.065	0.429

Character parameters of SCF samples.

Agilent 1100 liquid chromatograph, including LC solution working station, automatic sample injector and ultraviolet detector etc (Agilent technologies, USA). Zorbax Eclipse Plus C18 chromatographic column (4.6 × 250 mm, 5 μm, Agilent technologies, USA). AB135-S Electronic balance, 50-Bio UV-VIS spectrophotometer (Shanghai, China).

The reference substances include Schisandrol A (batch number: 14030307), Schisandrol B (batch number: 14030401), Schisantherin A (batch number: 14032110), Schisandrin A (batch number: 14031206), Schisandrin B (batch number: 14022106) and Schisandrin C (batch number: 103617) were purchased from Chengdu Biopurify Phytochemicals Ltd. and the purity greater than 98%. The ultrapurewater used in the experiments was prepared using a Milli-QUltrapure water purification system (Millipore, Bedford, MA, USA). Analytical-grade methanol was purchased from the Beijing, and other reagents were of analytical grade.

## Methods

### Ethical Statement

Investigate by the ethics committee of our hospital, the experiment design and experiment process is not involved ethical experiments.

## Study Design

This study included three steps. Step 1: Preliminary EQA for quality grade and weeding out of unqualified experts and samples; Step 2: Data modelling and designing EQA card; and Step 3: Validating the heritability of EQA among novices.

### Twice Judgement

#### Experts exclusion

Experts team consists of 13 experts, professors, and pharmacists who are engaged in the identification and quality research of traditional Chinese medicine. The information about those experts was collected in Table 2. They are from Peking University, Beijing Health Vocational College, China National Traditional Chinese Medicine Corporation, National Institutes for Food and Drug Control, Beijing Hospital of Traditional Chinese Medicine, Pharmacy department of Dongzhimen Hospital, Pharmacy department of Beijing Friendship Hospital, TCM Pharmacy of 302 Military Hospital, Pharmacy department of Beijing Jishuitan Hospital, Pharmacy department of Beijing Chinese Medicine Hospital, Pharmacy department of Guang’anmen Hospital, and so on. All of them have all worked for at least 15 years and accumulate a wealth of work experience.

**Table 2:** Experts Information.

Factors Experts	Years of career (years)	Work unit	Education background
a	32	Pharmacy	Modern
b	26	University	Modern
c	30	Quality control	Traditional
d	42	Pharmacy	Traditional
e	25	University	Modern
f	20	Pharmacy	Modern
g	40	Pharmacy	Traditional
h	51	University	Traditional
i	25	University	Modern
j	15	University	Modern
k	34	Pharmacy	Traditional
l	33	Quality control	Modern
m	30	Pharmacy	Traditional

### Delphi Survey

The Delphi survey is a type of investigation method proposed by American Rand Co. in the 1960s. This method can be applied to standardization research and various empirical evaluation index systems. At present, it is widely used in fields such as social science, medicine and health, and market decision making.

In this study, experts carried out a consulting activity by combining evaluation with a disordered sequence of assessment and final assessment. That is, experts assessed medical materials twice at different times. Notably, the medical materials evaluated were the same but were assessed in different orders. According to the survey results, we could analyse the reproducibility of individual experience and the consistency of group experience. A final assessment was then made after the evaluation. The flow chart of this process is shown in Fig. 7.

### Statistical Analysis of Delphi Survey

Error rate of individual (E_i_)

1 $${E}_{i}=\frac{{M}_{a}}{M}\times 100 \% $$

M_a_ represents the number of samples which get the different result by experts in twice judgments; M represents the total number of samples.

Error rate of Samples (E_s_)

2 $${E}_{s}=\frac{{N}_{e}}{N}\times 100 \% $$

N_e_ represents the number of experts make the different judgment on a same sample; N means the total number of experts.

Consistent rate of individual and group (C_ig_)

3 $${C}_{ig}=\frac{{M}_{c}}{{M}_{e}}\times 100{\rm{ \% }}$$

M_c_ represents the number of samples which get the same results between the expert individual and experts group. M_e_ represents the number of samples which get the same result by experts in twice judgments

Consensus of Samples (C_s_)

4 $${C}_{s}=\frac{{N}_{c}}{{N}_{a}}\times 100{\rm{ \% }}$$

N_c_ represents the number of experts in the self-reproduce expert who are consistent with the sample grade. N_a_ represents the total number of experts who can make the same judgment by themselves to a same sample.

Importance of the score (I_p_)

5 $${I}_{p}=\frac{{M}_{j}}{{M}_{n}}\times 100 \% $$

M_j_ represents the number of the experts who give the full mark. M_n_ means the number of experts. I_p_ was between 0 and 1, the bigger the value is, the greater the evaluation index is.

### HPLC analysis

The HPLC method was applied to test the content of six lignins include Schisandrol A (Sch A), Schisandrol B (Sch B), Schisantherin A (Schi A), Schisandrin A (Schisan A), Schisandrin B (Schisan B) and Schisandrin C (Schisan C) in SCF^25,26^.

Six lignans in different grades of SCF were analyzed on a HPLC system (Agilent 1100, Agilent technologies, USA) using a Zorbax Eclipse Plus C18 (4.6 × 250 mm, 5 μm). The column temperature was set at 35 °C and 10 μL of the standard or sample solution was injected onto the system. The mobile phase was composed of (A) methanol and (B) 0.5% aqueous acetic acid using a gradient program of 62% A for 0–25 min, 62–80% for 25–37 min, 80–90% for 37–50 min, with a mobile flow rate of 1.0 mL·min^−1^.

The mixed standard solution consisted of 0.1848 mL·mL^−1^ of Sch A, 0.1353 mL·mL^−1^ of Sch B, 0.1499 mL·mL^−1^ of Schi A, 0.1286 mL·mL^−1^ of Schisan A, 0.1412 mL·mL^−1^ of Schisan B and 0.1138 mL·mL^−1^ of Schisan C were prepared by adding an accurately weighed amount of each standard substance into volumetric flasks and dissolving them with methanol.

We accurately weighed 1 g of the powder and performed an extraction with 25 mL of methanol via ultrasonic extraction for 30 min. The extracted solution was cooled, contributing to weight loss during the extraction produce, and then filtered through a 0.22 μm micropore film to yield the sample solution.

The method validation was include precision, stability, repeatability, and recovery and the result was shows in Supplementary Table S1.

#### UV analysis

The UV method was used to test the contents of polysaccharides and polyphenols in different parts and different grades of SCF^27–29^.

Determination of polysaccharide content: 5 g of sample powder was accurately weighed, and extraction was performed with 50 mL distilled water and 2 h of refluxing. To the extract, 100 mL ethanol was added, and the mixture was left to stand for 12 h. Polysaccharide were filtered out and dissolved in 100 mL distilled water. A 1 mL aliquot was removed, and 1 mL distilled water, 1 mL 5% phenol and 5 mL concentrated sulfuric acid were added to an EP tube. The materials were mixed evenly and left to stand for 10 minutes. Water bath insulation was performed for 15 min at 40 °C, and then the sample was removed and cooled to room temperature. Ultraviolet spectrophotometry was performed to measure absorbance at λ = 490 nm.

Determination of polyphenol content: 5 g of sample powder was accurately weighed, and extraction was performed with 50 mL of methanol via ultrasonic extraction for 2 h. Approximately 0.5 mL of the extract was removed, and 2.0 mL 20% sodium carbonate buffer, 1.5 mL Folin-Ciocalteu colour reagent, and distilled water were added to create a total sample volume of 50 mL. The absorbance was measured at 760 nm in a water bath run for 1.5 h at 55 °C.

Method validation included evaluation of precision, stability, repeatability, and recovery. The results are shown in Supplementary Figure S1, Supplementary Tables S2 and S3.

#### Activity Determination

The hepatoprotective and anti-aging activities of the different grades of SCF were tested to explore their biological activity.

Hepatoprotective activity: 25 g of sample powder was accurately weighed, and extraction was performed with 250 mL of ethanol and 2 h of refluxing. The process was repeated twice, and the filtrate was combined with volatile ethanol and freeze dried. The different grades of SCF were dissolved with 0.5% CMC-Na physiological saline before gavage, and the final sample concentration was 10 g/mL. Bifendate was allocated to a 15 mg/mL solution with normal saline before use. CCl_4_ was mixed with 0.1% sterile olive oil solution before use, and the mixture was fully mixed.

Mice were randomly divided into 6 groups of 10 animals each. In the control group and CCl_4_-intoxicated group, animals were given a single daily dose of normal saline (20 mL/kg body weight) orally by gavage. In the test groups, animals were given a daily 20 mL/kg dose of SCF orally by gavage. All administrations were conducted over 7 days. On the 8th day, all mice except those in the control group were simultaneously given CCl_4_ (intraperitoneally, 20 mL/kg) 1 h after the last administration, while the control group received olive oil alone. Then, all animals were fasted for 24 h and were subsequently tested for the following analysis. Blood was collected from the eye. After blood collection, serum was separated by centrifugation at 3000 rpm at room temperature for 20 min. The serum ALT and AST values were measured with commercially available diagnostic kits.

Antioxidant activity: 800 μL of sample solution from samples of different concentrations were placed in a cuvette with 800 μL of a 150 μmol/L solution of DPPH radical added. Then, the mixture was shaken evenly and allowed to stand at room temperature in the dark for 30 min. Thereafter, the absorbance of the assay mixture was measured at 517 nm against a methanol blank using a spectrophotometer. DPPH radical scavenging capacity was expressed as the percentage inhibition of DPPH radical. The percentage inhibition of DPPH radical by SCF was calculated from the measured absorbance value according to the following equation:

6 $${\rm{Inhibition}}\,{\rm{of}}\,{\rm{DPPH}}\,{\rm{radical}}\,( \% )=({A}_{0}-{A}_{1})/{A}_{0}\times 100 \% $$

where A_0_ was the absorbance of the control (blank, without sample) and A_1_ was the absorbance in the presence of sample.

#### Statistical chemical analysis

SPSS 22.0 software was used for correlation analysis of the SCF samples and decision tree design. SIMCA-P^+^ 13.0 was used for PCA analysis, and Metaboanalyst (http://www.metaboanalyst.ca/) was used for clustering analysis. In the present study, PCA and clustering analysis were both performed on samples of various concentrations for all chemical contents and physical parameters. Prior to the analysis, all observations were normalized and saved as new variables. A *t*-test was performed for significance analysis.

### Data availability statement

All date were available at China Military Institute of Chinese Medicine, 302 Military Hospital.

## Electronic supplementary material

Supplementary Information
