# Supplementary material for: The Scientific Basis and Advantage of Human Experiential Assessment in the quality control of Chinese Herbal Medicines exampling as Schisandrae Chinensis Fructus
Source: Sci Rep. 2018 Apr 9;8:5695. doi: 10.1038/s41598-018-23619-5 (PMC5890249; doi:10.1038/s41598-018-23619-5)
Supplement: Supplementary file 1 — Supplementary Information [file 41598_2018_23619_MOESM1_ESM.docx]

**The Scientific Basis and Advantage of Human Experiential Assessment in the quality control of Chinese Herbal Medicines exampling as Schisandrae Chinensis Fructus**

Yongfeng Zhou^1†^, Dingkun Zhang^2†^, Haotian Li^1^, Haizhu Zhang^1^, Jixiang Fang^1^, Yanqin Ma^1^, Ping Zhang ^1^^*^, Jiabo Wang ^1*^, Xiaohe Xiao^1*^

1 China Military Institute of Chinese Medicine, 302 Military Hospital, Beijing, 100039, China

2 College of Pharmacy, Chengdu University of Traditional Chinese Medicine, Chengdu, 611137, China

***** Corresponding author

Ping Zhang: Email zhp1231@126.com

Jiabo Wang: Email pharm_sci@126.com

Xiaohe Xiao: Email pharmacy302xxh@126.com

† These authors contributed equally to this work.

|  | Repeatability  RSD/% | Stability  RSD/% | Precision  RSD/% | Linear Relationship | | RecoveryRSD/% |
| --- | --- | --- | --- | --- | --- | --- |
|  |  |  |  | Linear regression R | Linear range（μg/mL） |  |
| Schi A | 0.18 | 0.13 | 0.53 | y = 2x + 2 1 | 33.11~117.45 | 101.3 |
| Schi B | 0.36 | 0.21 | 0.33 | y=36.501x-5.7295 0.9998 | 27.21~98.11 | 100.5 |
| Schisant A | 0.17 | 0.30 | 0.92 | y=27.337x-9.601 0.9999 | 25.34~94.37 | 103.6 |
| Schisand A | 0.10 | 0.17 | 0.18 | y=38.387x-4.3676 0.9999 | 23.47~83.14 | 100.9 |
| Schisand B | 0.36 | 0.23 | 0.18 | y=29.421x-2.0095 0.9999 | 19.38~68.23 | 99.8 |
| Schisand C | 0.50 | 0.69 | 0.27 | y=24.531x-3.549 1 | 19.39~69.36 | 102.5 |

Table S1 The result of method validation


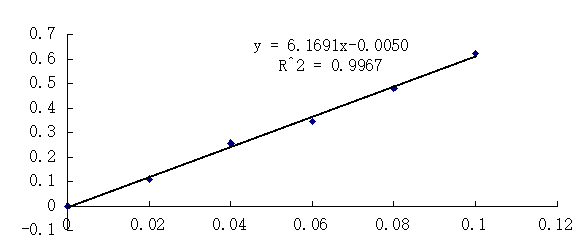


Fig. S1 The standard cruve about polysaccharide


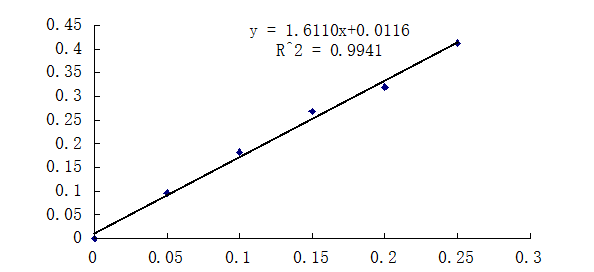


Fig. S2 The standard cruve about polyphenol

|  | Repeatability  RSD/% | Stability  RSD/% | Precision  RSD/% | Recovery  RSD/% |
| --- | --- | --- | --- | --- |
|  |  |  |  |  |
| polysaccharide | 1.08 | 2.14 | 2.58 | 3.31 |
| polyphenol | 3.25 | 2.64 | 3.10 | 2.58 |

Table S2 The result of method validation about UV method
